# Outcomes of Diabetes Management with Continuous Glucose Monitoring Technology

**DOI:** 10.26502/aimr.0243

**Published:** 2026-05-20

**Authors:** Daniel Vayser, Phoebe Wang, Nolan Dafesh, Devendra K. Agrawal

**Affiliations:** 1 Department of Translational Research, College of Osteopathic Medicine of the Pacific, Western University of Health Sciences, Pomona, California 91766 USA

**Keywords:** Automated insulin delivery, Continuous glucose monitoring (CGM), Dexcom, FreeStyle Libre, HbA1c, Hypoglycemia, Medtronic, Type 1 diabetes mellitus (T1DM), Type 2 diabetes mellitus (T2DM)

## Abstract

Diabetes mellitus is a chronic metabolic disorder that imposes a global economic burden. Type 1 diabetes mellitus (T1DM) results from autoimmune destruction of pancreatic β-cells, while type 2 diabetes mellitus (T2DM) is characterized primarily by peripheral insulin resistance with progressive β-cell dysfunction. Despite advances in pharmacologic and self-monitoring therapies, many patients fail to achieve recommended glycemic targets. Continuous glucose monitoring (CGM) has emerged as a transformative technology with the potential to address persistent gaps in diabetes management. This review critically synthesizes the current literature on CGM-associated clinical outcomes, cost implications, and technological limitations across major commercial platforms—Dexcom, Medtronic, and FreeStyle Libre—and examines future directions in CGM innovation. A comprehensive review of randomized controlled trials, prospective cohort studies, real-world registries, and meta-analyses was performed to evaluate CGM use in T1DM and T2DM populations across pediatric, adult, and older adult cohorts. Outcomes assessed included HbA1c, time in range (TIR), hypoglycemic events, healthcare utilization, cost, patient-reported outcomes, and adverse effects. CGM use was consistently associated with improvements in glycemic control, with HbA1c reductions ranging from approximately 0.4% to 1.5% across studies, and the greatest benefits observed in patients with higher baseline HbA1c. Hypoglycemic events were substantially reduced, including a 72% reduction reported in the HypoDE trial and reductions of up to 79% in the DIAMOND trial. CGM use was also associated with increased TIR, decreased glycemic variability, and reduced rates of diabetic ketoacidosis hospitalizations and emergency department visits, yielding meaningful cost offsets despite higher upfront device costs. Patient-reported outcomes—including treatment satisfaction, self-efficacy, and quality of life—improved consistently across studies. Among the major platforms, Dexcom systems demonstrated robust evidence in both T1DM and T2DM populations; Medtronic's MiniMed 780G achieved mean TIR values approaching 78.8% with optimal settings through automated insulin delivery; and FreeStyle Libre demonstrated particular value in T2DM populations on basal insulin or non-insulin therapy. Limitations included dermatologic complications, sensor lag during rapid glycemic change, alarm fatigue, accuracy variability across the hypoglycemic range, and procedural and mechanical issues. CGM technology has fundamentally reshaped contemporary diabetes care, with consistent and reproducible benefits across glycemic, economic, and patient-centered domains in both T1DM and T2DM populations. While important device-related and patient-centered limitations persist, the cumulative evidence support broader integration of CGM into routine diabetes management. Continued innovation in sensor accuracy, automated insulin delivery, and data analytics—combined with improved access and equitable reimbursement—will be essential to fully realize the potential of CGM for the growing global diabetes population.

## Introduction

Diabetes mellitus (DM) is a chronic metabolic disorder characterized by persistent hyperglycemia resulting from impaired insulin secretion, defective insulin action, or both. Dysregulation of endogenous glucose homeostasis leads to abnormalities in carbohydrate, lipid, and protein metabolism leading to the pathogenesis and exacerbation of many diseases [1–10]. Diabetes mellitus is broadly classified into two principal subtypes: Type 1 diabetes mellitus (T1DM) and Type 2 diabetes mellitus (T2DM).

**Figure 1:**
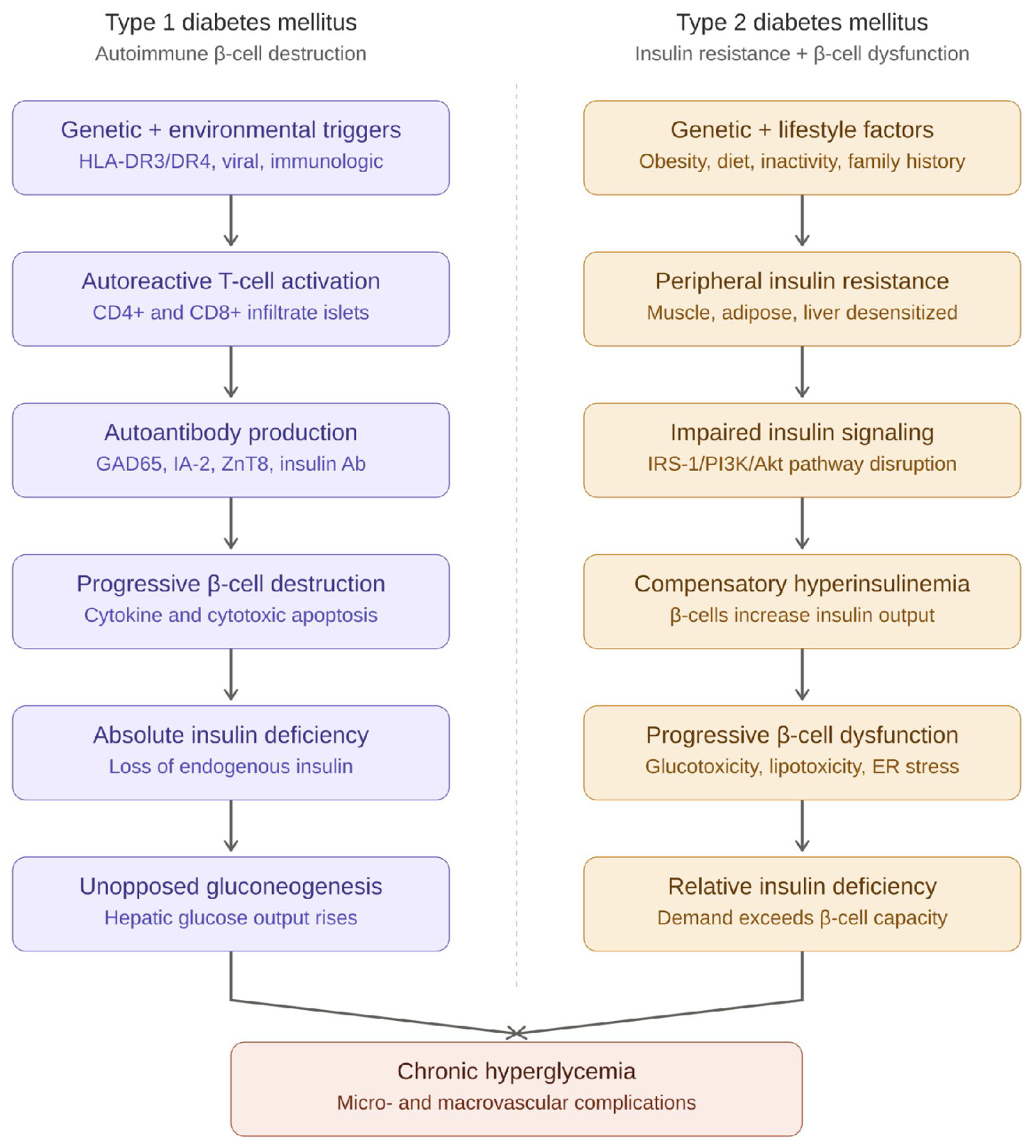
Cellular and molecular mechanisms underlying Type 1 and Type 2 diabetes mellitus. T1DM (left) is initiated by genetic susceptibility (HLA-DR3/DR4) and environmental triggers, leading to autoreactive T-cell activation, autoantibody production (GAD65, IA-2, ZnT8, insulin), progressive β-cell apoptosis, and absolute insulin deficiency with unopposed hepatic gluconeogenesis. T2DM (right) arises from genetic and lifestyle factors driving peripheral insulin resistance, impaired post-receptor signaling (IRS-1/PI3K/Akt), compensatory hyperinsulinemia, and eventual β-cell dysfunction from glucotoxicity, lipotoxicity, and ER stress, culminating in relative insulin deficiency. Both pathways converge on chronic hyperglycemia.

T1DM is an autoimmune condition characterized by immune-mediated destruction of pancreatic β-cells, resulting in absolute insulin deficiency. Consequently, patients are unable to produce endogenous insulin, impairing cellular glucose uptake and promoting hepatic gluconeogenesis, ultimately leading to chronic hyperglycemia. In contrast, T2DM is characterized primarily by peripheral insulin resistance, in which target tissues—including skeletal muscle, adipose tissue, and the liver—exhibit reduced responsiveness to insulin. Progressive β-cell dysfunction often develops over time, further exacerbating hyperglycemia and associated diseases and complications during and following surgery [11–20] (Figure 1).

Both T1DM and T2DM demonstrate multifactorial etiologies involving genetic predisposition and environmental influences. T1DM has a well-established genetic association with specific human leukocyte antigen (HLA) haplotypes, particularly HLA-DR3 and HLA-DR4. However, penetrance remains low; only approximately 0.5% of individuals harboring HLA-DR3 or HLA-DR4 mutations ultimately develop T1DM, and the disease may occur in individuals without these alleles [21]. These findings highlight the importance of environmental and immunologic triggers in disease pathogenesis (Figure 1).

T2DM also exhibits a strong hereditary component. A positive family history significantly increases risk, and concordance rates among monozygotic twins further support substantial genetic influence [22,23]. However, environmental and behavioral factors are critical contributors. T2DM has been strongly associated with obesity, urbanization, poor dietary patterns, air pollution exposure, and lower socioeconomic status [24]. The interplay between genetic susceptibility and modifiable lifestyle factors remains central to disease development.

Diabetes mellitus represents a major global public health challenge. Approximately 1 in 11 adults worldwide is affected, with nearly 90% of cases attributable to T2DM [11]. It is estimated that more than 600 million individuals will develop T2DM by 2030 [25]. T1DM incidence is similarly rising. Several studies report annual increases of 2–5% in regions including the Middle East, Europe, and Australia [26–28]. In the United States, T1DM incidence has increased across all demographic groups by approximately 2% per year [29]. The economic burden of diabetes is substantial. Global costs are estimated at $413 billion annually, with $306 billion attributed to direct medical expenditures [30]. These expenditures reflect not only pharmacologic management but also treatment of acute complications, long-term microvascular and macrovascular sequelae, and other adverse effects [31,32].

Prior to the discovery of insulin, diabetes mellitus was considered a fatal disease. The isolation of insulin by Frederick Banting, Charles Best, James Bertram Collip, and John Macleod fundamentally transformed disease prognosis [33]. In 1922, insulin was first successfully administered to a patient with T1DM, reversing what had previously been a terminal condition. Over the subsequent century, numerous therapeutic modalities have been developed for both T1DM and T2DM, including refined insulin analogs, self-monitoring blood glucose systems, metformin, sulfonylureas, glucagon-like peptide-1 (GLP-1) receptor agonists, insulin pumps, and continuous glucose monitoring (CGM) systems [31,32,34].

Since the first approval of CGM systems by the U.S. Food and Drug Administration in 1999, CGM technology has been associated with improved glycemic control, increased time in target range, reduced hypoglycemia, and improved patient-reported outcomes in both T1DM and T2DM populations [35]. These demonstrated benefits have contributed to rapid growth within the CGM market [36]. Currently, approximately 50% of individuals with T1DM and 12–13% of individuals with T2DM utilize CGM systems, including platforms developed by Dexcom, Abbott Laboratories (FreeStyle Libre), and Medtronic. While the expanding range of available devices increases patient choice, it may also introduce complexity for newly diagnosed individuals and clinicians determining optimal device selection.

Given the expanding body of literature surrounding CGM use, a comprehensive synthesis of available evidence is warranted. This review aims to critically evaluate the literature regarding CGM-associated clinical outcomes, cost implications, and technological limitations. Furthermore, it seeks to examine future directions in CGM innovation and the broader implications of these technologies for diabetes management.

### Overall Outcomes

Continuous glucose monitors (CGMs) have significantly improved the management of both Type 1 and Type 2 diabetes. Numerous studies have demonstrated that CGMs improve HbA1c levels, reduce healthcare costs, and increase patient engagement in diabetes self-management, particularly among pediatric populations. Additionally, CGM use has been associated with reductions in healthcare resource utilization, hypoglycemic events, and other diabetes-related complications. One of the most important indicators of successful diabetes management is HbA1c, making it a critical metric for evaluating the clinical effectiveness of CGM technology.

Despite advances in diabetes management, many patients in the United States continue to struggle to meet recommended HbA1c targets. Data from the T1D Exchange registry between 2016 and 2018 [37] demonstrated that only a small proportion of individuals achieved the glycemic goals recommended by the American Diabetes Association (ADA). Specifically, “the American Diabetes Association (ADA) HbA1c goal of <58 mmol/mol for youth was achieved by only 17% and the goal of <53 mmol/mol for adults by only 21%” [37]. However, the same analysis found that HbA1c levels were significantly lower among patients using CGMs compared with those who did not use CGMs (P < 0.001), even after adjusting for age, diabetes duration, race/ethnicity, and annual income. These findings suggest that CGM technology may play a critical role in helping individuals from diverse backgrounds achieve their glycemic targets and improve long-term clinical outcomes.

In addition to improving glycemic control, CGMs may also contribute to reductions in healthcare costs. A study conducted by Isaacson et al. [38] demonstrated that CGM use resulted in cost savings of approximately $417 per member per month among non-Medicare patients and $9 per member per month among Medicare patients. Although CGMs may initially appear more expensive due to device costs, long-term economic benefits associated with improved glycemic control and reduced complications may offset these expenses. For example, Wan et al. [39] reported that “the total 6-month costs were $11,032 (CGM) vs. $7,236 (control)” (P < 0.01). While CGM therapy may involve higher upfront costs, these expenses must be considered in the context of long-term reductions in diabetes-related complications and hospitalizations.

Reducing long-term complications associated with diabetes can also significantly decrease healthcare expenditures. Dennis et al. [40] demonstrated that total healthcare costs were significantly lower among patients using CGMs ($6,245) compared with those without CGMs ($7,786; t(698,086) = −71.41, P < 0.001). Furthermore, the cohort using CGMs experienced significantly fewer emergency room (ER) visits and inpatient (IP) hospital days at 3, 6, 9, and 12 months [40]. These reductions in healthcare utilization are likely associated with decreased incidences of diabetic ketoacidosis (DKA), hypoglycemia, or both. Similar findings were reported by Pablo Rodríguez et al. [41], who observed a 50% reduction in DKA hospitalization rates among CGM users (79.26 to 40.28 admissions per 10,000 person-years; rate ratio [RR]: 0.5 [0.40–0.63]) [41]. The most statistically significant reduction occurred among patients with HbA1c levels above 10%, with 136 fewer events per 10,000 person-years, resulting in hospital savings of €782,836.81 [41]. These findings further support the role of CGMs in reducing severe diabetes complications and associated healthcare costs.

Hypoglycemia represents one of the most dangerous complications of diabetes management. Symptoms can include palpitations, sweating, and tremors, and if left untreated, severe hypoglycemia can lead to seizures, loss of consciousness, or death. Numerous studies have demonstrated that CGM use significantly reduces hypoglycemic events. In a study conducted by Pratley et al. [42], hypoglycemic outcomes were compared between patients using CGMs and those relying solely on traditional finger-stick glucose monitoring. The study found that time spent with glucose levels below 70 mg/dL decreased from 73 minutes per day to 39 minutes per day among CGM users, representing an average reduction of 27 minutes of hypoglycemia per day [42]. The HypoDE study [43] was a German six-month randomized controlled trial that evaluated whether CGM use could reduce hypoglycemia incidence in patients treated with multiple daily insulin injections. The results demonstrated a 72% reduction in hypoglycemic events among CGM users compared with patients relying on traditional self-monitoring methods (incidence rate ratio 0.28 [95% CI 0.20–0.39], p < 0.0001) [43]. Similarly, the SILVER study demonstrated that CGM use reduced both moderate and severe hypoglycemia. Time spent in severe hypoglycemia (<54 mg/dL) decreased from 2.1% to 0.6% (P < 0.001), while time spent in moderate hypoglycemia (<70 mg/dL) decreased from 5.4% to 2.9% (P < 0.001) [44].

As previously noted, many individuals in the United States continue to struggle to achieve recommended HbA1c targets. Several factors may contribute to this challenge, including financial barriers and difficulties with diabetes self-management. Research has shown that CGM technology can improve patient engagement and self-efficacy in diabetes care. A study conducted by the American Diabetes Association’s Emily et al. [45] interviewed 47 patients using the FreeStyle Libre system and assessed the perceived impact of CGM technology on diabetes management. Among participants, 65% reported greater self-efficacy and increased engagement with their diabetes care. Similarly, Chang et al. [46] conducted a randomized controlled trial in which one group received real-time CGM devices combined with education based on self-regulation theory, enabling patients to independently adjust their health behaviors and diabetes management strategies. The control group received standard diabetes education focused on traditional self-monitoring of blood glucose. Researchers evaluated treatment adherence, diabetes self-efficacy, and glycemic control, finding that the CGM group demonstrated sustained improvements in self-efficacy, significant improvements in diabetes-related health behaviors, and reductions in HbA1c levels over time.

Quality of life has also been shown to improve among patients using CGMs. The study by Charleer et al. [47] conducted a FUTURE study with the American Diabetes Association involving 1,913 individuals with Type 1 diabetes who were provided CGM devices, with quality-of-life and metabolic outcomes assessed over a one-year period. The study reported significant improvements in treatment satisfaction compared with baseline and noted fewer absences from work. The DIAMOND Randomized Clinical Trial [48,49] was a prospective study involving 158 adults with poorly controlled Type 1 diabetes and evaluated quality-of-life outcomes among patients using CGMs compared with those using traditional self-monitoring of blood glucose. The study found that CGM users experienced significant reductions in diabetes-related distress and increased confidence in preventing hypoglycemia [48]. Similarly, the GOLD Randomized Clinical Trial [50], which included 161 adults with Type 1 diabetes, demonstrated that CGM users reported greater treatment satisfaction and improved subjective well-being compared with individuals using conventional glucose monitoring methods. Additionally, the JDRF Continuous Glucose Monitoring Trial [51], a multicenter randomized study involving 451 patients with Type 1 diabetes, reported high levels of patient satisfaction with CGM technology after 26 weeks of use [51].

The COMISAIR study [52] demonstrated that continuous glucose monitoring (CGM) is a major contributor to HbA1c reduction, independent of the insulin delivery method. This study compared three groups of patients with diabetes: a control group receiving standard therapy with multiple daily insulin injections and self-monitoring of blood glucose, a second group using an insulin pump without CGM, and a third group using both an insulin pump and CGM. The results showed that patients using both an insulin pump and CGM experienced a significant reduction in HbA1c levels, decreasing from 8.3% to 7.1%, compared with those using an insulin pump alone (P=0.0032). While both pump-based groups demonstrated significant improvements in HbA1c and reduced glycemic variability compared with the control group, only the cohort using both CGM and an insulin pump experienced a reduction in hypoglycemic events. These findings suggest that CGM plays a critical role in improving glycemic control and reducing complications in patients with diabetes, beyond the benefits provided by insulin pump therapy alone [52].

These findings are also consistent in patients with Type 2 Diabetes, a population that often struggles to maintain optimal glycemic control. The Steno2Tech Study [53] demonstrated that patients with Type 2 diabetes experienced significant therapeutic benefits from the use of continuous glucose monitoring (CGM). Over a 12-month period, patients using CGM showed a 15.2% increase in time spent within the target glucose range compared with the control group receiving standard care. Additionally, the CGM cohort experienced a greater reduction in HbA1c, decreasing by 0.9%, along with a reduction in mean glucose levels of 1.47 mmol/L compared with the control group. Patients using CGM also spent significantly less time above the target glucose range, with a 15.5% reduction over 12 months. Furthermore, the CGM group required 10.6 fewer units of insulin per day on average compared with the control group. Beyond glycemic outcomes, patients reported higher treatment satisfaction and lower levels of diabetes-related stress while using CGM technology. These findings suggest that CGM use can substantially improve glycemic control, reduce insulin requirements, and enhance quality of life in patients with Type 2 diabetes.

### Dexcom

**Figure 2:**
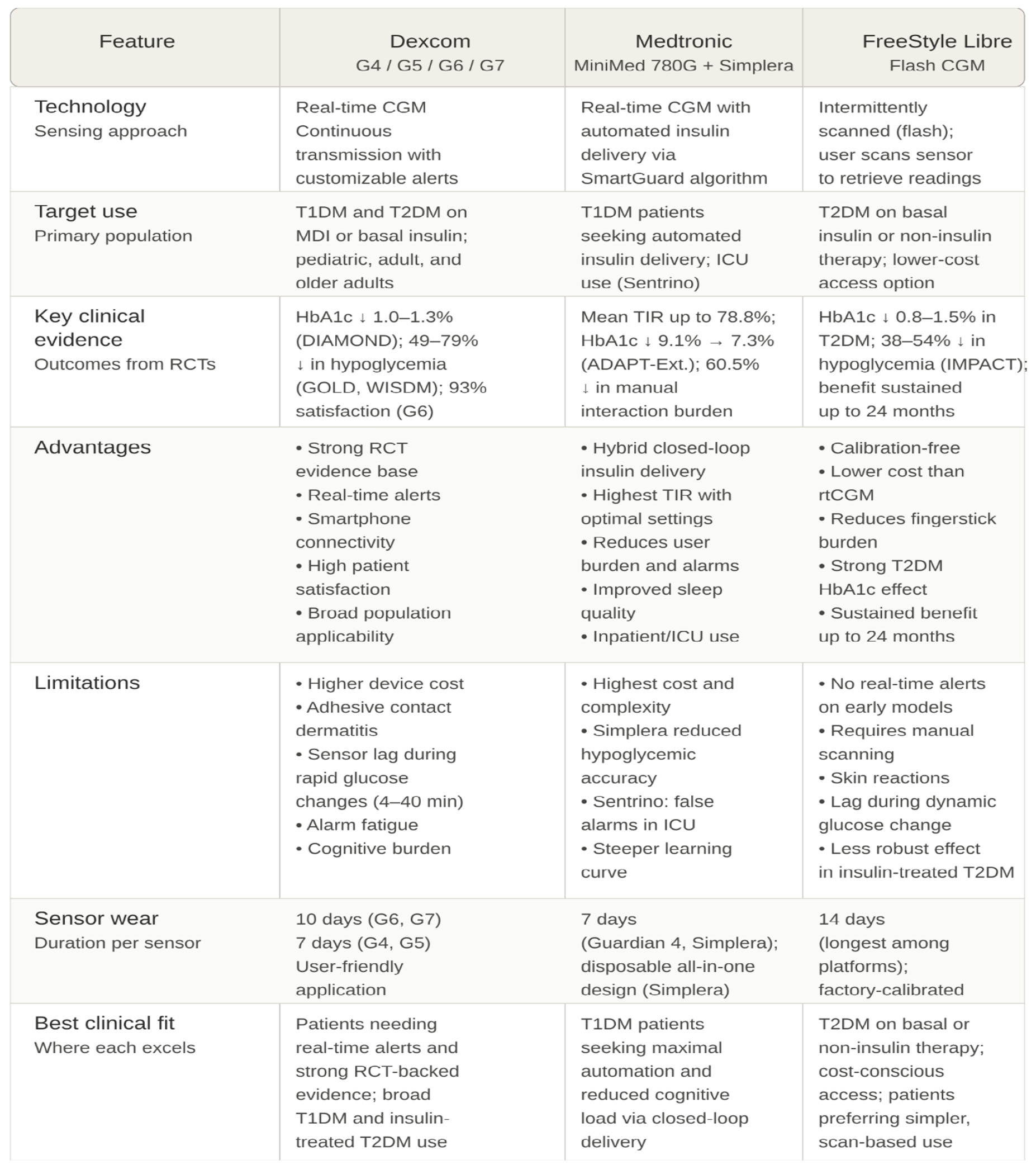
Comparative summary of leading continuous glucose monitoring platforms. Three commercial CGM systems—Dexcom, Medtronic (MiniMed 780G with Simplera CGM), and FreeStyle Libre—are compared across technology, target population, key clinical trial evidence, advantages, limitations, sensor wear duration, and best-fit clinical scenarios. CGM = continuous glucose monitoring; rtCGM = real-time CGM; MDI = multiple daily injections; T1DM/T2DM = type 1/2 diabetes mellitus; TIR = time in range; ICU = intensive care unit

Dexcom is one of the leading manufacturers of continuous glucose monitoring (CGM) systems and focuses on improving diabetes management through wearable sensors and data technology. Their major products include the Dexcom G7, G6, G5, and G4 systems. CGM use has been associated with reduced HbA1c, fewer hypoglycemic and hyperglycemic episodes, increased time in range, and improved quality of life (Figure 2).

The DIAMOND trial [48,49], a randomized controlled study examining the benefits of CGM use in adults with type 1 diabetes on multiple daily insulin injections, reported a 1.0% reduction in HbA1c in patients using the Dexcom G4 and G5 systems compared with a 0.6% reduction in the control group. Notably, 52% of participants using Dexcom experienced greater than a 1% HbA1c reduction, and participants with baseline HbA1c above 8.5% achieved a 1.3% reduction. Dexcom users also spent more time within the target glucose range (70–180 mg/dL), with 1.3 additional hours per day in range and a 4% reduction in hyperglycemic variability compared with controls. The CGM group experienced a 40% reduction in hyperglycemic events (>300 mg/dL; p<0.001), while the control group spent an additional 58 minutes in hyperglycemia. Hypoglycemic events were also substantially reduced, with 49%, 53%, and 69% reductions for glucose levels below 70, 60, and 50 mg/dL, respectively, and a 79% reduction in median time spent in hypoglycemia (<60 mg/dL) after 24 weeks.

The HypoDE study [43] corroborated these findings, reporting a 72% reduction in hypoglycemic events (p<0.0001) with the Dexcom G5, including significant reductions in nocturnal hypoglycemia between midnight and 6 a.m. The GOLD trial [50] further demonstrated that CGM use led to a 0.43% reduction in HbA1c compared with patients using traditional blood glucose meters, with three times as many CGM users achieving an HbA1c reduction greater than 1%, and an 80% reduction in severe hypoglycemic events, defined as events requiring assistance or resulting in unconsciousness.

The Wireless Innovation for Seniors with Diabetes Mellitus (WISDM) study [42] evaluated older adults with type 1 diabetes using the Dexcom G5 and found a median reduction in time spent in hypoglycemia from 5.1% to 2.7% over 5 months, while the control group showed no change. Time in the target range (70–180 mg/dL) increased by 8.8% (p<0.001), and mean HbA1c decreased by 0.3% (p<0.001). High adherence was observed, with more than 83% of participants wearing the device at least six days per week, indicating favorable user acceptance.

In patients with type 2 diabetes, CGM use has also demonstrated significant clinical benefits. Layne et al. [54] reported that adults using the Dexcom G6 or G7 spent 17.3% more time in range (70–180 mg/dL) over 12 months compared with baseline. Their glucose management indicator decreased by 0.5% (from 8.1% to 7.6%), and time spent above (>180 mg/dL) and below (<70 mg/dL) range decreased significantly. Device usage was high, with CGM worn 84.7% of days over one year, and activation of the high alert feature was associated with additional improvements in time in range. Martens et al. [55] found that adults with poorly controlled type 2 diabetes using basal insulin experienced a decrease in HbA1c from 9.1% to 8.0% over 8 months with CGM, compared with a reduction from 9.0% to 8.4% in the control group. The CGM cohort spent 59% of time in range (70–180 mg/dL) versus 43% in controls (p<0.001), with the percentage of time above 250 mg/dL reduced from 27% to 11%, and mean glucose decreased from 207 mg/dL to 179 mg/dL (p<0.001).

Gilbert et al. [56] similarly demonstrated that CGM use improved outcomes in both type 1 and type 2 diabetes, with mean HbA1c decreasing from 8.2% to 7.1% (p<0.001). More than half of participants with baseline HbA1c above 7% achieved reductions greater than 1%. Participants also reported decreased diabetes-related stress and fewer concerns regarding hypoglycemia. Overall, 93% of participants were satisfied or very satisfied with the G6 system, and 73% found it very easy to use.

### Medtronic

Medtronic is a leading manufacturer with diabetes technologies, giving a focus to automated insulin delivery (AID) with continuous glucose monitoring to give the best outcomes in outpatient clinic and hospital settings. Their star technology, the MiniMed 780G system, uses a SmartGuardTM algorithm to make insulin delivery automated, and this has shown notable differences in glycemic control, including less burden with manual management, lessened HbA1c and an increased Time in Range (TIR) [57–59]. Looking at diverse populations, from pediatric populations in the United Kingdom to multiple cohorts in Europe, this system has helped most users obtain their clinical targets [57,60,61].

The clinical efficacy of the 780G system is emphasized. In the ADAPT-Extension Study, where an absolute TIR increase of 29.7% [58] was noted when patients had switched from multiple daily injections (MDI) to the 780G system. The mean of the HbA1c in this group had decreased from 9.1% to 7.3% within a time frame of 6 months [58]. Taking data from 100,000 users, these findings were strengthened even more, with an overall mean TIR of 72.3% and a GMI of 7.0% [57] being reported. When users used the “optimal settings” like 100mg/dL glucose target and a 2-hour active insulin time, the results were emphasized even more. This pushed the mean TIR to 78.8% in large scale analyses [62] (Figure 2).

Medtronic’s sensor portfolio also consists of next generation Simplera SyncTM and SimpleraTM . These sensors are designed to be friendly to users, as well as being calibration free [63,64]. Applications in the real world with SimpleraTM sensor with 780G system had shown a mean TIR of 73.1% accuracy with low rates of hypoglycemia [63]. Referencing the SUCCEED trial, the Simplera SyncTM sensor allowed youths to reach a 71.4% TIR and adults to reach an 80.2% [64]. While the SimpleraTM sensor gave high levels of accuracy with euglycemia (MARD of 10.5%), some studies noted that it could have lower accuracy in the hypoglycemic Rangel and a higher rate with early sensor failures compared to newer generation CGMs [64,65].

For more high acuity environments, the Medtronic Sentrino system was noted for being able to provide CGM for ICU patients who were critically ill. A large strength with the Sentrino System was its ability to detect asymptomatic hypoglycemia that is commonly missed by intermittent point-of-care testing, being able to identify 94.3% of hypoglycemic events [66]. It is a very reliable system, being noted that it can reach up to 98.3% of data “on time” [66]. It does have limitations, though, as noted by its “alarm fatigue” among nurses due to there not being enough large scale randomized controlled trials showing a definitive reduction with hospital length of stay compared to standard care or mortality, as well as high false alarm rates [67,68].

Going past Medtronic’s advanced systems glycemic metrics, there is a significant reduction in the daily psychosocial burden of diabetes. Going to the 780G system with the GuardianTM 4 sensor showed a reduction in manual interactions and “burden area” by 60.5%, mainly through removing routine finger-prick calibrations, as well as decreasing system interruptions [59]. This gave an increased quality of life to users and caregivers. It also decreased diabetes related stress and improved sleep quality because of the automation of nighttime insulin delivery and a decrease in nocturnal alarms [69,70].

### FreeStyle Libre

FreeStyle Libre is a leading-edge flash continuous glucose monitoring (CGM) system that improves diabetic management through intermittently scanned glucose data and reduced reliance on fingerstick monitoring. FreeStyle Libre CGM use has been correlated with reduced HbA1c, fewer hyperglycemic and hypoglycemic episodes, and increased time in range (TIR). These have subsequently led to reduced healthcare utilization and an improved quality of life (Figure 2).

Utilization of FreeStyle CGM significantly improves HbA1c levels, especially for type 2 diabetics. The retrospective chart review study and meta-analysis conducted by Carlson et al. [71] revealed that FreeStyle Libre continuous glucose monitoring allows for clinically significant HbA1c reductions in type 2 diabetic adults on basal insulin, with a decrease of −1.1% to −1.4% over 3-6 months. Similarly, the study conducted by Wright et al. [72] demonstrated a larger reduction of −1.5%, with type 2 diabetic patients with a baseline HbA1c ≥12% having reductions of up to −3.7%, indicating a strong baseline-dependent effect. These findings are also supported by Kröger et al. [73], who showed consistent HbA1c reductions of ~−0.8-0.9% across multiple European cohorts, and Elliot et al. [74], who reported a −0.8% decrease in a Canadian real-world population.

Though HbA1c is significantly lowered primarily for Type 2 diabetics, both type 1 and type 2 diabetics benefit HbA1c levels that are lowered with sustained duration. This is supported by the Evans et al. [75] meta-analysis of over 30,000 patients across 75 studies, which showed consistent lowering of HbA1c levels of ~0.4-0.6% in both type 1 and type 2 diabetics, as well as lasting effects for up to 24 months. There were especially greater improvements in patients who began with a higher baseline HbA1c. Eeg-Olofsson et al. [76] additionally confirmed sustained HbA1c reductions (−0.44% in T1DM and −0.66% in T2DM) can remain for over 9-15 months after initiation of FreeStyle Libre CGM use.

Randomized trials in type 1 diabetics revealed both better glycemic control and safety following use. Leelarathna et al. [77] demonstrated a −0.8% HbA1c reduction with Libre in comparison to −0.2% in controls, as well as a +130 min/day increase in TIR and a −43 min/day reduction spent in hypoglycemia through the FLASH-UK trial for these patients. Bolinder et al. [78] conducted an IMPACT trial, which demonstrated that Libre could assist in achieving a 38% reduction of time spent in hypoglycemia, with even greater reduction at severe thresholds and in nocturnal periods. It also reduced variation in the glycemic state and TIR without worsening HbA1c. Of note, real-world data from Tyndall et al. [79] indicated that there was especially significant HbA1c reductions ranging from −0.4% to −1.3% overall in patients with poorly controlled Type 1 diabetes, as well as an increase in achieving personal goals and lowering DKA admission rates. Even in the pediatric population, Campbell et al. [80] demonstrates that there is improved TIR of +0.9 hours/day with modest HbA1c reductions (~−0.4%) and reduced hyperglycemia.

For non-insulin treated or mild type 2 diabetic populations, Libre can have lasting benefit. Wada et al. [81] showed that usage improved TIR (+2.36 hours/day), lowered hyperglycemia (−2.66 hours/day), improved glucose variability and maintained HbA1c improvement even after discontinuation. In the same population, Brown et al. [82] conducted an IMMEDIATE study that also showed improved TIR of 76.1% compared to 64.3%, reduced hyperglycemia, and an even stronger HbA1c reduction of −0.9% compared to −0.5% with Libre paired with education. Similarly, Yaron et al. [83] found a greater HbA1c reduction of −0.82% vs −0.33%, along with improved goal attainment without increased hypoglycemia risk.

In Type 2 diabetics receiving insulin treatment, Libre primarily benefits by providing additional safety. The REPLACE trial conducted by Haak et al. [84] did not find an overall HbA1c difference compared to fingerstick monitoring, but revealed substantial improvement in hypoglycemia, including a 43% reduction in time spent at <70 mg/dl, and a 54% loss in nocturnal hypoglycemia. Despite the lack of change in HbA1c, Charleer et al. [47] also demonstrated that Libre aided in a ~50% reduction of severe hypoglycemia and hypoglycemic coma, as well as reduced work absenteeism and improved treatment satisfaction. Deshmukh et al. [85] supported Libre’s safety benefits further by revealing less diabetes-related distress, reduced hospital utilization, and improved hypoglycemia awareness.

Aside from glycemic metrics, improved clinical outcomes are also seen in real-world populations. Guerici et al. [86] found fewer DKA and severe hypoglycemia in Type 2 diabetics receiving baseline insulin; these significantly contributed to reduced acute diabetes event (ADE) related hospitalizations from 2.01% to 0.75% at 12 months, and 0.60% at 24 months (~63-70% reduction). Even for Type 2 diabetics with more advanced disease on prandial insulin, Bergenstal et al. [87] demonstrated that acute diabetes events were reduced by 61% and a 32% decrease in all-cause hospitalizations, while Miller et al. [88] revealed similar improvements even in both basal insulin and noninsulin-treating populations (−32% ADE, −15% hospitalizations). The FLARE-NL registry by Fokkert et al. [89] of real-world type 1 and type 2 Libre users further showed improved quality of life, lowered disease burden, fewer hospital admissions (~65%), and less work absenteeism (~60%).

Overall, FreeStyle Libre provides rapid benefit (within ~3 months) of durable HbA1c reduction, improved time in range, lowered hypoglycemia, improved patient engagement, and reduced fingerstick burden, with real-world evidence also demonstrating lowered acute diabetes-related events and hospitalization rates.

Study limitations include studies being predominantly observational and retrospective in design, which limit the strength of causal inference; incomplete reports of glycemic metrics in some datasets as well as adherence; and reliance on claims data in larger studies. Device-related limitations include skin limitation, adverse reactions to adhesive, interstitial glucose lag time, potential sensory accuracy variability, and possible psychological burden resulting from continuous glucose visibility.

### Adverse Effects of CGM

Despite the well-established clinical benefits of continuous glucose monitoring (CGM), its use is associated with several potential adverse effects and limitations that warrant consideration. These include dermatological complications, technical limitations, safety and device reliability, patient-centered barriers, and procedural and mechanical issues.

Dermatological complications are the most common adverse effects seen with continuous glucose monitoring use and are a primary driver of device discontinuation. Rather than the sensing mechanism itself, prolonged exposure to device adhesives can cause adverse reactions, such as irritant and allergic contact dermatitis. Foti et al. [90] identified acrylate compounds, especially isobornyl acrylate, as key allergens that could precipitate allergic contact dermatitis in CGM users. Heinemann et al. [91] reported that adverse cutaneous reactions often included pruritus, induration, and erythema following prolonged use. Svedman et al. [92] conducted a cross-sectional analysis that revealed a substantial proportion of patients would have to discontinue or switch devices secondary to clinically significant skin reactions. Additional studies have indicated that dermatologic complaints are common, with approximately 25-30% of users experiencing skin reactions in study cohorts, and real-world populations reporting higher rates closer to 40% [92,93]. These findings demonstrate that CGM-associated skin complications are primarily material-driven and can become a significant barrier to long-term use.

In addition to dermatological complaints that can result from CGM use, the device itself is inherently limited in its temporal accuracy due to both device-related and physiologic factors. As CGMs indirectly measure glucose levels in the bloodstream by recording interstitial fluid levels, device-reported values will differ from plasma glucose levels due to physiologic and sensor-related delay. Beyond physiologic factors, Davey et al. [94] showed that the lag can also be attributed to the sensor itself, with delays ranging from 8.3 to 40.1 minutes depending on the rate of glucose change, and that there can be even greater discrepancies during rapid glycemic fluctuations. Sinha et al. [95] also support that CGM delays are multifactorial and result from both physiological diffusion and instrumentation, causing delays ranging from approximately 4 to 11 minutes across devices and patient populations. These delays are responsible for reduced accuracy, especially when glucose levels rapidly fluctuate. Delays are further exacerbated in dynamic conditions. Zaharieva et al. [96] showed that CGM values can lag by approximately 12 ± 11 minutes during aerobic exercise, and that mean absolute relative difference (MARD) can increase as high as 13-22%. Overall, CGM performance is limited during rapid glycemic change, where both physiologic diffusion delay and intrinsic sensor lag lead to clinically significant differences between interstitial and blood glucose levels (Figure 2).

CGM systems can also raise safety, and reliability concerns due to variability in sensor performance, accuracy limitations, and device longevity. Eichenlaub et al. [97] demonstrated that meaningful performance variability exists between devices and can impact clinical interpretation, with reported MARD values ranging from approximately 9% to over 16%. Similarly, Luijf et al. [98] showed that CGMs are not sufficiently accurate for fully autonomous use, with manufacturers recommending confirmatory capillary glucose measurements before treatment decisions, particularly in hypoglycemic ranges. In addition to accuracy limitations, sensor performance may degrade over time, with studies demonstrating inconsistencies between controlled clinical environments and real-world home use. Wu et al. [99] further illustrate that CGM performance is vulnerable to both physiologic and environmental interference, such as temperature changes, biofouling, and electroactive substances, all of which can reduce signal stability and lead to measurement error with prolonged use. Combined, these findings highlight that while CGMs provide valuable trend data, limitations in long-term stability, accuracy, and consistency introduce important safety considerations in clinical practice.

Even with the clinical benefits of CGM, patient-centered barriers such as alarm fatigue and cognitive burden may limit device adherence. Polonsky and Hessler [100] showed that though CGM improves glycemic outcomes, patients often were deterred from continuous use due to frustration with frequent alerts and the psychological burden of persistent data monitoring. Tanenbaum et al. [101] also indicated that CGM use can exacerbate anxiety in diabetics who have frequent alarms or perceive constant pressure to respond to rapid glucose fluctuations in real time. Messer et al. [102] further support that patients with frequent alerts triggered by inaccurate or rapidly fluctuating glucose levels can experience alarm fatigue, causing desensitization to alerts or disabling of notifications altogether, reducing the safety advantages of the device. Though CGMs provide valuable real-time information, the continuous alerts and stream of data can paradoxically worsen cognitive and emotional burden and generate a significant barrier to optimal device utilization.

Procedural and mechanical challenges may also significantly compromise device reliability and sustained use. Markov et al. [103] revealed that real-world users can often experience interruptions in CGM use due to signal loss, insertion difficulties, or sensor detachment, which can create a gap in glucose data collection. Krouwer [104] further illustrates that device malfunctions are the most commonly reported CGM-related issue, surpassing patient injury events and underscoring the importance of mechanical reliability in overall device performance. Aside from potential interruptions in data collection, procedural complications can also arise. Regulatory safety evaluations by the U.S. Food and Drug Administration [105] indicate that CGM insertion and removal carry risks including pain, bleeding, infection, and retained sensory fragments. These mechanical and procedural limitations can disrupt continuous data acquisition and impact derived metrics such as time-in-range, ultimately reducing the reliability and clinical utility of CGM systems in real-world practice.

## Discussion

This review synthesizes contemporary evidence on continuous glucose monitoring (CGM) systems in the management of Type 1 (T1DM) and Type 2 (T2DM) diabetes mellitus, evaluating clinical, economic, and patient-centered outcomes across the major commercial platforms—Dexcom, Medtronic, and FreeStyle Libre. Taken collectively, the literature demonstrates that CGM technology has become a transformative component of contemporary diabetes care, with consistent and clinically meaningful benefits across a broad spectrum of patient populations. However, important limitations persist, and several barriers to optimal implementation remain unresolved.

Across the studies reviewed, three central themes emerge. First, CGM use is consistently associated with improved glycemic control, as evidenced by reductions in HbA1c, increased time in range (TIR), and decreased glycemic variability. The DIAMOND, GOLD, HypoDE, COMISAIR, and Steno2Tech trials—spanning both T1DM and T2DM populations—demonstrate that these benefits are reproducible across diverse clinical contexts, including patients on multiple daily injections, basal insulin, and insulin pump therapy [43,48,49,50,52,53]. The magnitude of HbA1c reduction appears to be greatest in patients with higher baseline values, a pattern observed across all three major device platforms [49,55,106,107]. This baseline-dependent effect is clinically important, as it suggests that the patients who stand to benefit most from CGM technology are precisely those with the poorest glycemic control.

Second, CGM use is associated with substantial reductions in hypoglycemic events, including severe and nocturnal hypoglycemia. The HypoDE trial reported a 72% reduction in hypoglycemic events, while the SILVER and WISDM studies demonstrated similar benefits in moderate and severe hypoglycemia [42,43]. These findings are particularly meaningful given that hypoglycemia represents one of the most feared and dangerous complications of insulin therapy, with potential consequences including seizures, loss of consciousness, and death. The capacity of CGM to reduce hypoglycemia-related morbidity may be among its most clinically significant contributions, particularly for older adults and patients with impaired hypoglycemia awareness.

Third, CGM technology yields meaningful improvements in patient-reported outcomes, including treatment satisfaction, diabetes-related distress, self-efficacy, and quality of life. The DIAMOND, GOLD, JDRF, and FUTURE studies consistently report that CGM users experience reduced diabetes-related distress and improved confidence in self-management [47–51]. These psychosocial benefits are not merely ancillary; they likely contribute to sustained engagement with diabetes self-care behaviors, which in turn reinforces the glycemic improvements observed.

While the three major CGM platforms—Dexcom, Medtronic, and FreeStyle Libre—share core clinical benefits, they differ meaningfully in design philosophy, integration capabilities, and target use cases. Dexcom systems (G4 through G7) have demonstrated robust performance in randomized trials and real-world settings, with strong evidence supporting their use in both T1DM and T2DM populations on diverse insulin regimens [49,54–56] (Figure 2). Medtronic’s MiniMed 780G system, paired with the SmartGuard^™^ algorithm, represents a distinct approach centered on automated insulin delivery (AID), and large real-world datasets from over 100,000 users demonstrate mean TIR values approaching 72–79% with optimized settings [57,62]. FreeStyle Libre, as an intermittently scanned (flash) CGM, occupies a unique position in offering a lower-cost, calibration-free option that has demonstrated value in T2DM populations on basal insulin or non-insulin therapy, where HbA1c reductions of 0.8% to 1.5% have been reported [106–113].

Direct head-to-head comparisons between platforms remain limited, and most existing literature evaluates devices in isolation rather than against one another. Consequently, device selection in clinical practice is often driven by factors beyond comparative efficacy, including cost, insurance coverage, integration with insulin delivery systems, sensor wear duration, and individual patient preferences. Future comparative effectiveness research is needed to better inform device selection, particularly as new sensor generations such as Medtronic’s Simplera^™^ and Simplera Sync^™^ enter the market [63,64].

The economic case for CGM use is increasingly well-supported. Although device acquisition costs are substantial, multiple analyses indicate that these expenses are offset by reductions in diabetes-related complications, emergency department utilization, and inpatient hospitalizations [38,40,41]. The 50% reduction in diabetic ketoacidosis hospitalizations reported by Pablo Rodríguez de Vera Gómez et al. [41] translated into significant institutional cost savings, and Guerci et al. [86] similarly demonstrated reductions in acute diabetes event hospitalizations of approximately 63–70% over 24 months following FreeStyle Libre initiation [86]. These findings carry important implications for healthcare policy and reimbursement, as they support the economic sustainability of broader CGM access. However, the cost-effectiveness of CGM remains heterogeneous across patient populations, with the greatest economic benefits observed in patients at highest risk for severe hypoglycemia or DKA.

Despite the encouraging body of evidence, several important limitations warrant consideration. From a methodological standpoint, much of the available real-world data is observational and retrospective in design, which limits causal inference and raises concerns regarding selection bias—patients who adopt CGM may differ systematically from non-adopters in ways that independently influence outcomes. Many studies also rely on claims-based data or registry analyses with incomplete capture of glycemic metrics and adherence patterns. Randomized controlled trials, while methodologically stronger, are often limited by relatively short follow-up periods and selected populations that may not reflect the broader diabetes population.

From a technological standpoint, CGM systems remain subject to important performance limitations. Interstitial-to-plasma glucose lag times ranging from 4 to 40 minutes, particularly during periods of rapid glycemic change or aerobic exercise, can introduce clinically meaningful discrepancies between device-reported and actual blood glucose values [47,95,96]. Sensor accuracy, as measured by mean absolute relative difference (MARD), continues to vary across devices and is particularly limited in the hypoglycemic range [97,98]. Environmental factors, biofouling, and electroactive interference may further degrade sensor performance over prolonged use [99]. These limitations underscore that CGM remains an adjunct to, rather than a replacement for, clinical judgment, and that confirmatory capillary measurements remain warranted in certain circumstances.

Patient-centered limitations also merit attention. Dermatological complications, particularly contact dermatitis related to acrylate-based adhesives, affect a substantial proportion of users and represent a leading cause of device discontinuation [90–93]. Alarm fatigue, cognitive burden, and the psychological weight of continuous data exposure can paradoxically undermine the benefits CGM is designed to confer [100–102]. Procedural and mechanical issues—including signal loss, sensor detachment, insertion difficulties, and rare adverse events such as retained sensor fragments—further compromise the reliability of continuous data acquisition [103–105]. Addressing these limitations through improved adhesive formulations, refined alert algorithms, and enhanced sensor durability represents an important priority for future device development.

The cumulative evidence supports a broader role for CGM in routine diabetes management, extending beyond traditional indications in T1DM to include patients with T2DM on insulin and, increasingly, those on non-insulin therapies. Clinicians should consider CGM not merely as a glucose measurement tool, but as a behavioral intervention that promotes patient engagement, self-efficacy, and informed therapeutic decision-making. Patient selection, education, and ongoing support remain critical determinants of success, as device benefits depend substantially on sustained and informed use. Attention should be directed toward populations at highest risk for hypoglycemia, those with elevated HbA1c, and those who demonstrate motivation for active self-management—groups in which the available evidence suggests the greatest clinical and economic returns.

Several promising avenues warrant continued investigation. The integration of CGM with automated insulin delivery systems, exemplified by the Medtronic MiniMed 780G, represents a significant step toward closed-loop diabetes management and continues to evolve toward fully autonomous systems. The development of next-generation sensors—including calibration-free, factory-calibrated, and disposable platforms—promises to further reduce user burden and expand accessibility. Inpatient and critical care applications of CGM, while currently limited by alarm fatigue and a paucity of large, randomized trials demonstrating mortality benefit, represent an area of active development [65–67,106]. Finally, the integration of CGM data with artificial intelligence and machine learning platforms holds the potential to enable predictive glycemic modeling, personalized therapeutic recommendations, and earlier identification of patients at risk for adverse outcomes.

## Conclusion

Continuous glucose monitoring has fundamentally reshaped the landscape of diabetes care over the past two decades. The accumulated evidence demonstrates consistent benefits across glycemic, economic, and patient-centered domains in both T1DM and T2DM populations, with effects that are reproducible across major commercial platforms and a wide range of clinical contexts. While important limitations—including device-related accuracy constraints, dermatological complications, alarm fatigue, and methodological gaps in the existing literature—merit continued attention, the overall trajectory of evidence supports the expanded integration of CGM into routine diabetes management. Continued innovation in sensor technology, automated insulin delivery, and data analytics, combined with improved access and equitable reimbursement, will be essential to fully realize the potential of CGM to improve outcomes for the growing global population affected by diabetes mellitus.

## Key Points

- Diabetes mellitus affects approximately 1 in 11 adults worldwide, with nearly 90% of cases attributable to T2DM and a global annual economic burden of approximately $413 billion, of which $306 billion reflects direct medical expenditures.
- Despite advances in diabetes care, only 17% of youth and 21% of adults with T1DM in the United States achieve American Diabetes Association HbA1c targets, underscoring the persistent need for improved monitoring and management strategies.
- CGM use is consistently associated with clinically meaningful HbA1c reductions ranging from approximately 0.4% to 1.5% across both T1DM and T2DM populations, with the greatest benefits observed in patients with higher baseline HbA1c.
- CGM substantially reduces hypoglycemic events, with reductions of 72% in the HypoDE trial and up to 79% in the DIAMOND trial, providing critical safety benefits particularly for older adults and patients with impaired hypoglycemia awareness.
- CGM use yields meaningful economic value through reduced diabetic ketoacidosis hospitalizations (up to 50%), fewer emergency department visits, and lower acute diabetes event rates, offsetting higher upfront device costs over time.
- Patient-reported outcomes—including treatment satisfaction, diabetes-related self-efficacy, and quality of life—improve consistently with CGM use, supporting its role as both a clinical and behavioral intervention.
- Each major platform serves a distinct clinical niche: Dexcom offers strong RCT-backed real-time monitoring across diverse populations, Medtronic's MiniMed 780G enables hybrid closed-loop automated insulin delivery with mean time in range approaching 78.8%, and FreeStyle Libre provides a calibration-free, lower-cost option particularly valuable in T2DM.
- Important limitations of CGM technology persist, including dermatologic complications affecting up to 25–40% of users, sensor lag of 4–40 minutes during rapid glycemic change, accuracy variability in the hypoglycemic range, and patient-centered burdens such as alarm fatigue.
- Most existing real-world evidence is observational and retrospective, limiting causal inference; further randomized comparative effectiveness research is needed to inform optimal device selection and long-term outcomes.
- Future directions include expanded integration of CGM with automated insulin delivery, calibration-free and disposable sensor platforms, broader inpatient and critical care applications, and the integration of CGM data with artificial intelligence for predictive glycemic modeling and personalized care.
